# Inducing illusory control ensures persistence when rewards fade and when others outperform us

**DOI:** 10.3758/s13423-020-01745-4

**Published:** 2020-05-18

**Authors:** Bettina Studer, Shawn N. Geniole, Maike L. Becker, Christoph Eisenegger, Stefan Knecht

**Affiliations:** 1 Institute of Clinical Neuroscience and Medical Psychology, Medical Faculty, University of Düsseldorf, Düsseldorf, Germany; 2 Department of Neurology, Mauritius Hospital Meerbusch, Meerbusch, Germany; 3 Neuropsychopharmacology and Biopsychology Unit, Faculty of Psychology, University of Vienna, Vienna, Austria; 4 Social-Neuroendocrinology Laboratory, Department of Psychology, Nipissing University, North Bay, Ontario Canada; 5 Department of Psychology, University of the Fraser Valley, Abbotsford, Canada

**Keywords:** Behavior enhancement, Illusion of control, Agency, Persistence, Motivation

## Abstract

Persisting even when the rewards of continued effort are fading is essential for achieving long-term goals, skills, and good health, alike. Yet, we often quit when things get hard. Here, we tested whether augmenting the feeling of control through external measures increases persistence under such discouraging circumstances. In two laboratory experiments, we first induced illusory control by manipulating the base-rate of positive outcomes and then tested the effect of this elevation of participants’ perceived control upon their persistence under diminishing returns and in a competition against a stronger opponent. Induced illusory control significantly enhanced people’s persistence in both of these motivationally challenging situations. Our findings demonstrate that motivation is dependent upon perceived, rather than objective, control, and reveal that this can be leveraged to counteract quitting behavior when things get hard, for instance in rehabilitation, physical activity interventions, or other training settings.

**Electronic supplementary material:**

The online version of this article (10.3758/s13423-020-01745-4) contains supplementary material, which is available to authorized users.

## Introduction

Persisting, even in the absence of reward or progress, is rightfully idealized in our society, as it is essential for achieving long-term goals. For instance, acquiring a new skill, maintaining good health, and regaining lost motor and cognitive functions after brain injury all require intensive training across multiple, repetitive sessions. Yet, particularly when the expected immediate reward is small, our brains’ valuation system is likely to signal that the effort of continuing is not worth it (Bailey, Simpson, & Balsam, 2016; Chong et al., 2017). Early quitting is a frequent phenomenon in physical activity and rehabilitation interventions and drastically hampers their effectiveness. Participants of a physical activity intervention against obesity missed their 10,000 steps a day goal by an average of 4,000 steps (Adams et al., 2013), merely 43% of the absolvents of New Zealand’s ‘exercise on prescription’ intervention reached the minimum target of 150 min of physical activity a week (Lawton et al., 2008), and stroke patients prescribed 30 min of self-directed daily rehabilitative training exercised for only 5−15 min (Tyson et al., 2015). How can individuals be enticed to persist with an activity for longer?

We propose that this may be achieved by inflating perceived personal control during an activity. Perceived personal control refers to the belief that outcomes are influenced by our behavior rather than determined by chance or an external force. Consistent with Expectancy Value Theories of motivation (e.g., Eccles & Wigfield, 2002; Lawler & Porter, 1967; Vroom, 1964) and Self-Efficacy Theory (Bandura, 1977; Bandura & Locke, 2003), we reason that perceived personal control co-determines the expectancy of achieving a wanted instrumental outcome and thereby motivation. Feeling in control over one’s environment has furthermore been theorized as inherently rewarding (Eitam, Kennedy, & Tory Higgins, 2013; Karsh, Eitam, Mark, & Higgins, 2016; Nafcha, Higgins, & Eitam, 2016), and might influence performance expectancy, which in turn has been postulated to act on goal-action coupling and focus attention unto the task (Wulf & Lewthwaite, 2016). Finally, we reason that a sense of personal control is also a prerequisite for feelings of competence, which govern intrinsic motivation according to Self-Determination Theory (Deci & Ryan, 2000; Ryan & Deci, 2000, 2007). Intriguingly, extant experimental research showed that perceived control is susceptible to manipulation of the task environment. Frequent delivery of positive outcomes (Alloy & Abramson, 1979; Gillan et al., 2014; Jenkins & Ward, 1965; Tobias-Webb et al., 2017), near-misses (Clark, Studer, Bruss, Tranel, & Bechara, 2014), and provision of choice all induce an “Illusion of Control” (IoC) (Langer, 1975), where a person’s feeling of personal control is exaggerated compared to the true contingency between outcomes and their actions or choices (Presson & Benassi, 1996; Stefan & David, 2013). This suggests that inducing illusory control could be a viable mechanism to enhance motivation to perform an activity and thereby persistence with that activity (see also Studer & Knecht, 2016). Yet, even though the IoC can be observed in multiple real-life contexts (Fenton-O'Creevy, Nicholson, Soane, & Willman, 2003; Pronin, Wegner, McCarthy, & Rodriguez, 2006; Rogers, 1998) and has been subject to extensive theoretical consideration (Langer, 1975; Thompson, Armstrong, & Thomas, 1998; Thompson et al., 2004), its potential to increase goal-directed behavior and motivation has not yet been systematically explored by the empirical behavioral sciences.

Here, we tested whether induced illusory control evoked longer subsequent persistence in two different motivationally challenging situations and samples of human volunteers (N=70 and N=99). Both experiments used new laboratory tasks that model real-life training conditions temporally, socially, and physically, employed a manipulation of the density (i.e., base-rate) of the positive outcome to elicit low or high levels of illusory controls, and probed the effect of induced illusory control upon persistence. Task persistence is not only a well-established behavioral array of motivation (see, e.g., Chong, Bonnelle, & Husain, 2016; Der-Avakian, Barnes, Markou, & Pizzagalli, 2016; Kirkden & Pajor, 2006; Patall, Cooper, & Robinson, 2008), but also constitutes an outcome-modulating intervention target in clinical and non-clinical training settings. Experiment 1 investigated persistence in the context of diminishing rewards, simulating the typical training situation where progress is initially fast but then slows. Experiment 2 tested persistence in a rigged social competition, where the opponent was stronger and persistence even led to monetary losses, i.e., simulating situations where others outperform and beat us.

## Methods

### Participants and procedures

#### Experiment 1

Seventy healthy adults took part in a 60-min testing session (29 males, 41 female, *M*_age_ = 51 years, *SD*_age_ = 15, range = 29−79), and were randomly allocated to one of three between-subject task conditions. They were reimbursed €8 per hour plus a variable bonus dependent on task earnings, ranging between €0.10 and €4.50 (*M* = €3.90). All participants gave written informed consent, and the experiment was approved by the independent ethics committee at the University of Düsseldorf (protocol # 5120).

#### Experiment 2

Experiment 2 was conducted in a new sample of 99 adult males (*M*_age_ = 26.15 years, *SD*_age_ = 6.83, range = 18−65), randomly allocated to one of two between-subject conditions. The sample was restricted to men due to being part of a larger protocol examining links between male sex hormones, cognition, and behavior. Participants received €10 or course credits, plus their earnings on the experimental task (*M* = €2.70, range = €0.00−€4.09). All participants gave written informed consent, and the experiment was approved by the independent ethics committee at the University of Vienna (protocol # 1918/2015). Participants were tested in a group session (three to ten individuals), in separate cubicles of a large room.

#### Sample-size determination

Given that these are the first experiments testing effects of induced illusory control upon persistence, no precedent for expected effect sizes existed. However, we reasoned that to be relevant for clinical and non-clinical applications, elicited between-condition differences in persistence would have to be of moderate effect size, at least. The sample sizes of our experiments allow detecting effects of *f* ≥ .38 and *d* ≥ .57 with a power of .80, respectively.

### Tasks

#### Experiment 1

**Fig. 1:**
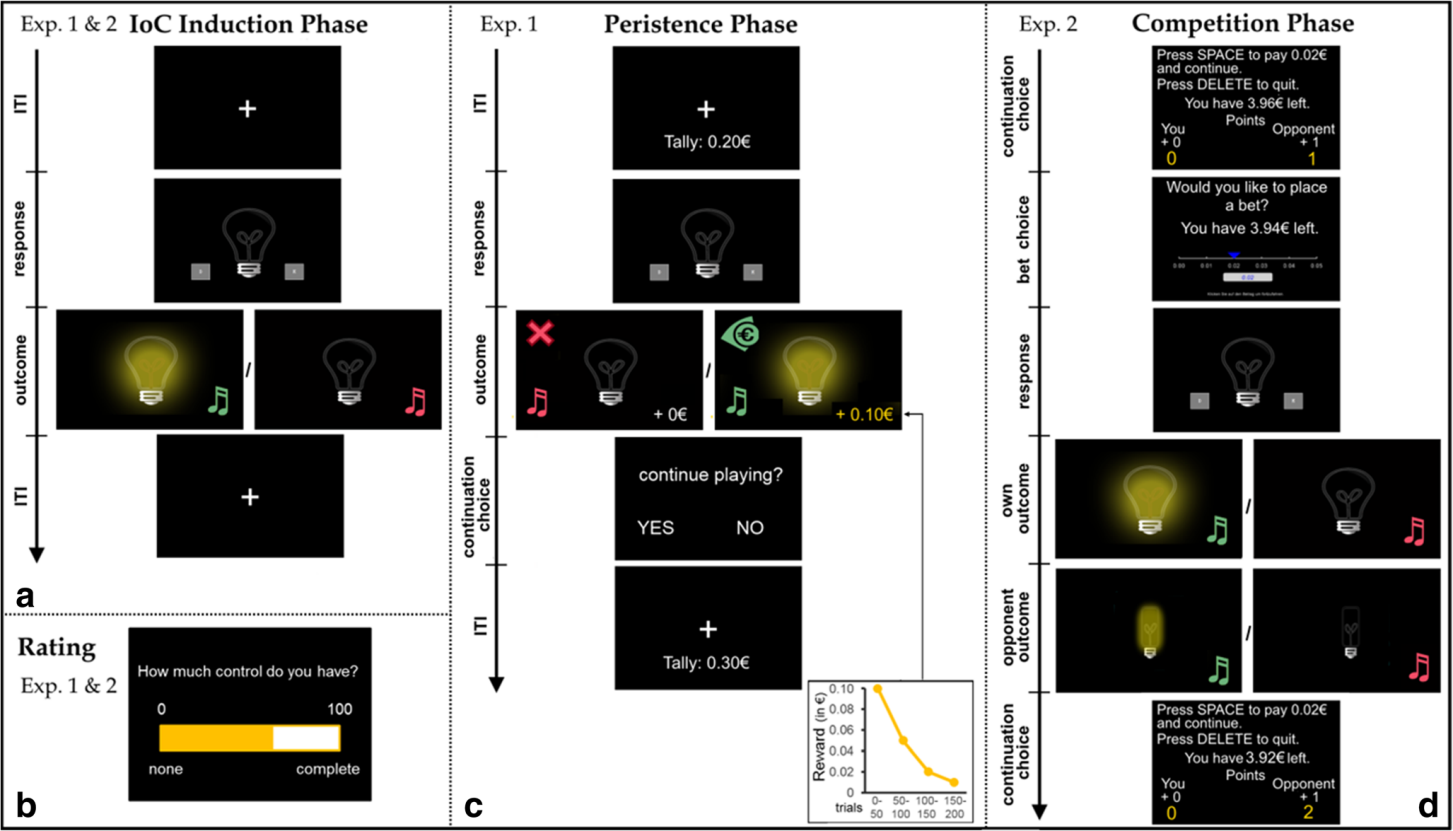
Experimental paradigms. (**A**) Illusion of Control (IoC) induction phase (used in both experiments): On each trial, participants selected between two possible response options, and then saw either the positive outcome (light bulb on) or the negative outcome (light bulb off). (**B**) Participants rated their personal control on a 0–100% visual analog scale, after each trial block. (**C**) Persistence phase of Experiment 1: Participants received a progressively decreasing monetary reward for each positive outcome (light bulb on) and were asked on each trial whether they wanted to persist with or quit the task (see Methods for further details). (**D**) Competition phase of Experiment 2: On each trial, participants first decided whether to play on or leave the competition. If they decided to remain in the competition, they next chose a bet (0−5 cents) and then made a binary choice with the aim of illuminating their light bulb. Once they saw their own outcome (normal light bulb on or off), the opponent's outcome (squared light bulb on or off) was displayed and the competition score and participants’ task earnings were updated (see *Methods* for further details). Musical notes, cross, and € pictograms were added for illustration purposes and did not appear on the task screen; original texts were in German. *ITI* intertrial interval, *Exp.* experiment

Our newly developed paradigm entailed an IoC induction phase followed by a subsequent persistence test with progressively diminishing rewards (see Fig. 1A and B). During the IoC induction phase, portrayed to participants as a learning phase, a light bulb was presented, and subjects were instructed to attempt to illuminate this light bulb by pressing one of two response buttons.^1^ Next, the light bulb either illuminated (positive outcome) or remained off (negative outcome). Unbeknown to subjects, illumination of the light bulb was always non-contingent, i.e., independent of which response button was pressed. To evoke different levels of perceived control, the base-rate of the positive outcome – henceforth termed “outcome density” − was varied across three between-subject conditions, with the light bulb illuminating in 70% (“high density”), 50% (“medium density”), or 30% (“low density”) of trials. No explicit information about reward likelihood, contingencies, or the existence of multiple experimental conditions was given to participants; instead, they were simply instructed to try to illuminate the light bulb as often as possible. This IoC induction phase consisted of three blocks of 30 trials each, and at the end of each block, participants rated their personal control over the light bulb illumination on a visual analog scale ranging from 0% to 100% (0% = “NO CONTROL, the lighting of the bulb had nothing to do with my button choices and but was entirely random”; 50% = “MEDIUM CONTROL, my button choices had some impact on the lighting of the bulb, but I did not have complete control”; 100% = “COMPLETE control, the lighting of the bulb was completely determined by my button choices”).

In the persistence phase, portrayed as the real play phase, participants received a monetary reward every time the light bulb illuminated, with the amount decreasing exponentially over successive trial blocks (from €0.10 to €0.01), such that performing the task became increasingly less attractive. Participants were informed about this progressively diminishing pay-out scheme after having completed the IoC induction phase and immediately before starting the persistence phase, and were told that they could terminate the task and cash in their winnings whenever they wanted. After each trial, they decided whether to end or continue the task. The number of trials completed before quitting served as the main persistence measure. During this phase, outcome density was set to 50% for all participants, such that monetary reward rate was identical for all participants and potential differences in persistence could be unequivocally attributed to the preceding IoC induction. The persistence phase included a maximum of four blocks of 50 trials each.

#### Experiment 2

Participants first again played an unrewarded version of the IoC task, this time without knowing that lightbulb illuminations would be associated with financial earnings later. This first phase again served to induce a high or low IoC, with outcome density being set to 75% (“high density”) and 25% (“low density”) across two between-subject conditions. Six blocks of 20 trials each were administered, and ratings of control were collected after each block. After completing this IoC induction phase, participants were told that they would play the task again, but this time compete against an anonymous – and in truth fictitious – opponent (see Fig. 1C). In each trial, both the participant and the opponent tried to illuminate their light bulb and whoever succeeded won. A non-recoverable 2 cent entry fee and rigging of the outcome densities (participant = 50%, opponent = increasing progressively from 50% to 80%) made competing costly and disadvantageous. Neither light bulb illumination nor competition outcomes were contingent on the participants’ selected responses. Critically, participants were free to quit the competition with their accumulated monetary winning, and instead continue playing by themselves without monetary rewards, whenever they wanted. Number of completed competition trials served as the main outcome measure. In addition, participants could wager up to 5 cents on each competition round. If they won, they received double the wager back; if there was a draw, the original wager was returned; if they lost, the wager was lost. Average bet amount served as a secondary outcome measure. Further, participants rated their own control and that of the opponent at the beginning of the competition, after each block of trials, and at the end of the competition.

Subjects were also administered personality questionnaires to allow us to explore potential trait influences on ratings of control (see Supplementary Material).

### Statistical analyses

First, we verified that our manipulation of outcome density successfully modulated perceived control by conducting mixed repeated-measures ANOVAs of control ratings during the IoC induction phase with the predictors Outcome Density Conditions (three and two levels, respectively) and Block (three and six levels, respectively). Next, we tested for induced control-mediated and direct effects of this manipulation on subsequent persistence, through a mediation model with the linear response variable “number of completed trials” serving as the outcome measure, Outcome Density Condition during the preceding IoC induction phase as the predictor (Experiment 1: 30%, 50%, or 70%, Experiment 2: 25% or 75%), and individuals’ control ratings at the end of the IoC induction phase as the mediator. In Experiment 1, average observed persistence was higher than expected, with 48 participants completing the maximum number of trials. This truncation induced a significant positive skew in the persistence measure. Therefore, we also conducted a supplementary equivalent mediation model with the binary outcome variable early termination (0 = yes, 1 = no) as a (reversed) persistence measure.^2^ In Experiment 2, average bet amount and final earnings served as secondary outcome measures and were also investigated with equivalent models.

Statistical analyses were computed in SPSS and R, and are reported two-sided with alpha set at .05. Given the between-subject designs and high within-group variances, outlier diagnostics were conducted for all analyses. In cases where influential outliers were found, the results of robust analyses calculated with the “robustbase” (Maechler et al., 2018) and “MASS” R packages (Venables & Ripley, 2002) are reported, following recent recommendations (Field & Wilcox, 2017). Due to a lack of robust methods for mediation analyses with categorical predictors, influential outliers (cooks distance > 4 * mean) were removed prior to those analyses calculated with the “lavaan” R package (Rosseel, 2012) (Exp.1: n=2 (persistence), n = 2 (early termination); Exp. 2: n=9 (persistence), n=8 (payout), n=3 (betting)).

## Results

### Induction of illusory control

**Fig. 2:**
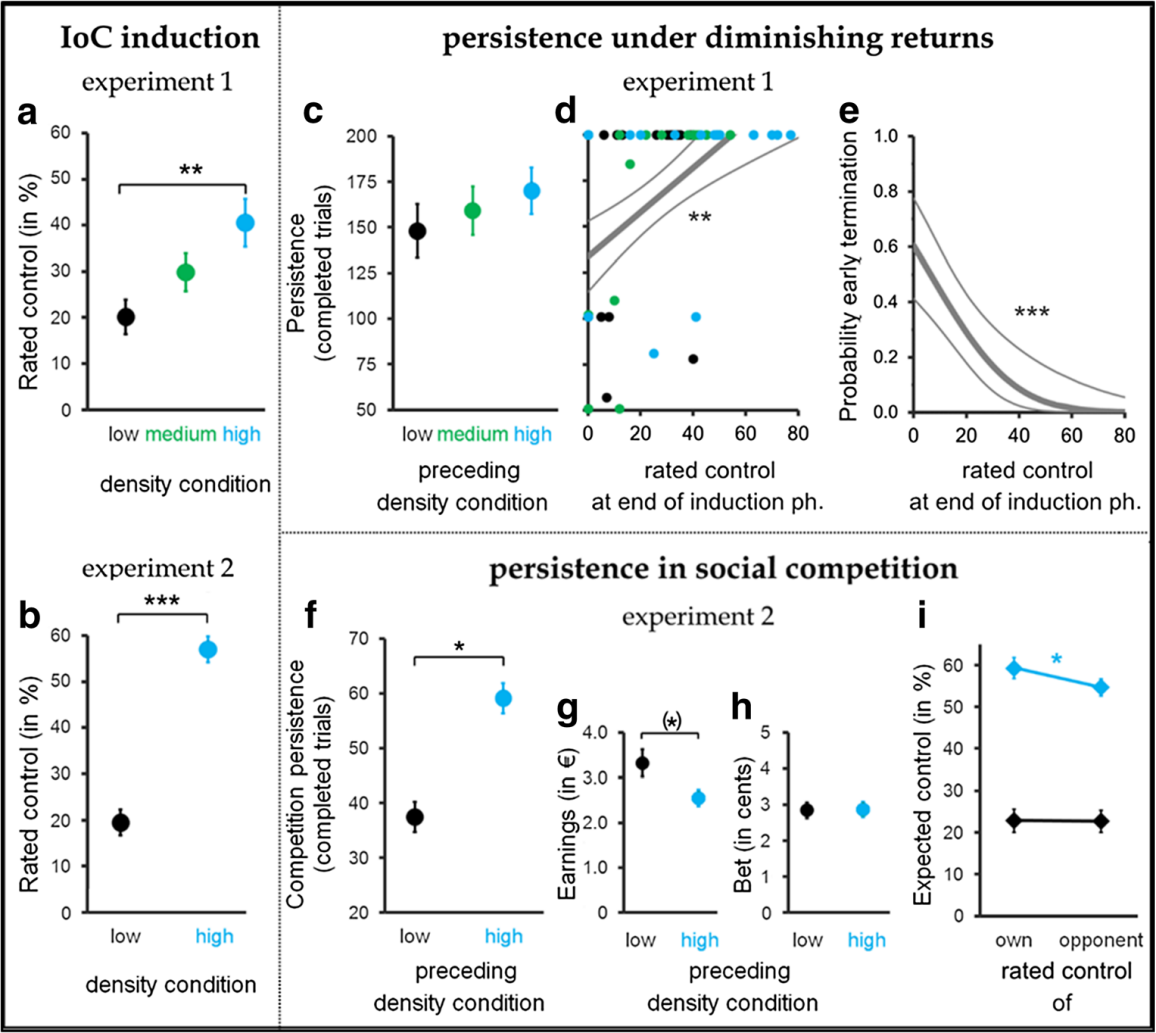
Induction of illusory control and effects upon persistence. (**A** and **B**) Induced illusory control increased significantly as a function of the outcome density condition in both Experiment 1 (**A**) and Experiment 2 (**B**). (**C**–**E**): Experiment 1 − Induced illusory control enhanced persistence under diminishing returns. Rated control at the end of the Illusion of Control (IoC) induction phase mediated the effect of the preceding outcome density condition on persistence (**C**), with persistence increasing (**D**) and probability of early termination (**F**) decreasing with higher levels of induced control. Thick lines in D and E indicate the regression lines, fine lines the 95% CI, restricted to the range of observed values. (**F**−**I**): Experiment 2 − Induced illusory control increase persistence in a disadvantageous competition. Observed mean competition persistence (**F**), task earnings (**G**), and average bets (**H**) and predicted control ratings taken before the start of the competition (**I**) are shown as a function of density condition in the preceding IoC induction phase. Error bars represent SEM. ***, ** and * denote significant differences with *p* ≤ .001, *p* ≤ .01, and *p* ≤ .05, respectively, (*) denotes a difference with p ≤ .1

Participants’ ratings of their control over the light-bulb illumination confirmed that the outcome density manipulation successfully induced illusory control in a dose-dependent manner. In both experiments, ratings of personal control during the IoC induction phase differed significantly between the outcome density conditions (Experiment 1: *F* (2, 67) = 5.552, *p* = .006, *η*_*p*_^*2*^ = .14, Experiment 2: *F* (1, 97) = 92.27, *p* < .001, *η*_*p*_*2* = .49), with participants allocated to higher density conditions experiencing more illusory control (see Fig. 2A and B).

### Evoked illusory control prolongs persistence under diminishing returns (Experiment 1)

Experiment 1 tested whether evoked illusory control was associated with increased subsequent persistence under diminishing return. Those who had undergone the high-density condition during the IoC induction phase completed 169 persistence trials on average, the medium-density group 159 trials, and the low-density group 148 trials (see Fig. 2C). Mediation analysis confirmed that the density manipulation significantly affected subsequent persistence through its effect on perceived control, *B(SE)* = 14.39 (5.50), *z* = 2.62, *p* = .009, proportion mediated effect = 0.79 (see Table 1). On average, persistence grew by 12 trials with each 10-point increase in rated control at the end of the IoC induction phase, *B(SE)* = 1.25 (0.30), *z* = 4.13, *p* < .0001 (see Fig. 2D). No significant independent effect of the outcome manipulation was found, *B(SE)* = 3.94 (8.93), *z* = 0.44, *p* = .65. Equivalent results were obtained for the binary reverse persistence measure early termination (see Table 1). Probability of early termination decreased significantly with higher levels of induced control, *B(SE)* = -0.010, *z* = -4.27, *p* < .0001, on average by 4% with each 10-point increase in rated control (see Fig. 2E), and induced illusory control significantly mediated the effect of the density condition upon probability of early termination of the subsequent persistence phase, *B(SE)* = -0.112(0.043), *z* = -2.63, *p* = .009, proportion mediated effect = 0.98.

**Table 1:** Experiment 1: Results of mediation models of persistence, betting, and task earnings

	Β	SE	95% CI	z	p
Persistence					
indirect effect (condition -> control -> persistence)	14.39	5.50	[3.62, 25.18]	2.62	.009
condition -> perceived control	11.54	3.22	[5.23, 17.86]	3.59	<.001
perceived control -> persistence	1.25	0.30	[0.66, 1.84]	4.13	<.001
direct effect (condition -> persistence)	3.94	8.93	[-13.57, 21.44]	0.44	.66
model R^2^ = .24					
Early termination					
indirect effect	-0.112	0.043	[-0.196, -0.029]	-2.63	.009
condition -> perceived control	11.55	3.22	[5.233, 17.857]	3.59	<.001
perceived control -> early termination	-0.010	0.002	[-0.014, -0.005]	-4.27	<.001
direct effect	-0.002	0.07	[-0.141, 0.137]	- 0.03	.98
model R^2^ = .24					

*Note:* The magnitude of the regression coefficient for the effect of condition on perceived control varies across models due to the varying number of (removed) influential outliers

### Evoked illusory control prolongs persistence in a social competition (Experiment 2)

Experiment 2 tested whether evoked illusory control also manifested in increased persistence in a costly social competition. Participants who experienced the high-density condition during the preceding IoC induction phase remained significantly longer in the competition, *Β(SE)* = 17.56 (7.66), *z* = 2.29, *p* = .02 (see Fig. 2F), and tentatively ended the contest with less money, *Β(SE)* = -.40 (.22), *z* = -1.85, *p* = .06 (see Fig. 2G), than those who had undergone the low-density condition. Neither of these effects of our manipulation were significantly mediated by individuals’ ratings of perceived control, ps ≥ .41 (see Table 2). However, the high-density condition group also predicted having a higher control than their opponents before starting the competition, *MD*_trimmed_ = 4.60%, 95% CI = [1.22, 7.98], *Yuen t*(29) = 2.78, *p*_corr_ = .018, whereas those in the low-density condition forecasted their opponent as having a similar degree of control to themselves, *MD*_trimmed_ = 0.16%, 95% CI = [-4.23, 4.55], *Yuen t*(30) = 0.08, *p*_corr_ = .99 (see Fig. 2I). This suggests that high-evoked illusory control caused participants to enter the competition overconfidently. Bets placed did not vary systematically as a function of previous outcome density condition or perceived control (see Fig. 2H and Table 2).

**Table 2:** Experiment 2: Results of mediation models of persistence, betting, and task earnings

	Β	SE	95% CI	z	p
Persistence					
indirect effect (condition -> control -> persistence)	-2.69	3.29	[-9.14, 3.76]	-0.82	.41
condition -> perceived control	29.98	5.75	[19.06, 40.92]	5.38	<.0001
perceived control -> persistence	-0.09	0.11	[-0.30, 0.12]	-0.08	.41
direct effect (condition -> persistence)	17.56	7.66	[2.54, 32.58]	2.29	.02
model R^2^ = .05					
Task earnings					
indirect effect	-0.05	0.10	[-0.25, 0.14]	-0.54	.59
condition -> perceived control	30.44	5.49	[19.69, 41.19]	5.46	<.0001
perceived control -> earnings	-0.002	0.003	[-0.008, 0.005]	-0.55	.58
direct effect	-0.40	0.22	[-0.82, 0.02]	-1.85	.06
model R^2^ = .05					
Average bet					
indirect effect	0.25	0.20	[-0.14, 0.65]	1.26	.21
condition -> perceived control	34.94	4.97	[25.19, 44.67]	7.03	<.0001
perceived control -> bet	0.007	0.006	[-0.004, 0.018]	1.32	.19
direct effect	-0.25	0.35	[-0.94, 0.44]	-0.72	.47
model R^2^ = .01					

*Note:* The magnitude of the regression coefficient for the effect of condition on perceived control varies across models due to the varying number of (removed) influential outliers

## Discussion

Our results attest that elevating perceived control enhances persistence when rewards fade and things get hard. Our illusory control-inducing manipulation led participants to subsequently persist longer under diminishing returns (Experiment 1) and in a disadvantageous, costly social competition (Experiment 2). Furthermore, in Experiment 1, individuals’ level of induced illusory control directly and significantly predicted their subsequent persistence. These results empirically validate the theory that motivation is dependent upon our feeling of personal control. With some variation in terminology and precise proposed mechanism, this assumption is common to multiple well-regarded psychological theories of motivation, including Expectancy Valence Theories (Eccles & Wigfield, 2002; Lawler & Porter, 1967; Vroom, 1964), Self-Efficacy Theory (Bandura, 1977; Bandura & Locke, 2003), and Self-Determination Theory (Deci, 1980; Deci & Ryan, 2000; Ryan & Deci, 2000, 2007), yet has been subjected to surprisingly few causal empirical explorations. Arguably the strongest previous empirical support stems from laboratory gambling research showing that near-miss outcomes elicit a higher wish to continue gambling (Clark et al., 2014) and longer actual play (Côté, Caron, Aubert, Desrochers, & Ladouceur, 2003) than full losses. The current research advances this previous work in several critical ways. First, as verified by the collected ratings, we directly manipulated participants’ perceived personal control and did so in a graded manner. Second, we showed that individual differences in the strength of induced illusory control translated to individuals’ persistence in Experiment 1. Third, and most importantly, we tailored our experiments to test persistence under motivationally challenging situations with direct relevance to potential applications in real-life training, rehabilitation, and intervention settings. In Experiment 1, we employed a progressively decreasing incentivization schedule, where longer persistence was beneficial to the participant but where the rewards for continued action gradually shrank, to stimulate a typical skill-learning situation where gains are initially large but then diminish. And in Experiment 2 we showed that illusory control evokes overconfidence and prolongs persistence in a rigged social competition. Although these effects were detrimental to financial earnings in the set-up we used, they show great promise for buffering against discouragement in other real-world interactions where opponents may be stronger or making faster progress, but persistence is nonetheless beneficial. Indeed, fear of potential discouragement through unfavorable social comparison stops many healthcare professionals from using competition, forgoing its recently verified training-enhancing effects (Studer, Van Dijk, Handermann, & Knecht, 2016).

In both experiments, extrinsic motivation arguably progressively diminished during the persistence phase, due to the gradually decreasing rewards in Experiment 1 and to the increasingly frequent competition losses in Experiment 2. Even though these extrinsic rewards were matched across experimental groups, our results show that induced illusory control through our outcome density manipulation was able to shift participants’ “motivational breakpoint,” i.e., the point when they quitted the task. What could be the precise mechanism underlying this persistence-enhancing effect? We argue that induced illusory control augmented individuals’ expectancy that they would achieve the wanted outcome (a rewarded lightbulb illumination in Experiment 1 and a competition win in Experiment 2) through their actions and thereby the subjective motivational value of continued action (cf. Studer & Knecht, 2016). This interpretation aligns with Expectancy Valence Theories (Eccles & Wigfield, 2002; Lawler & Porter, 1967; Vroom, 1964), which postulate that motivation is determined by an individual’s belief about their personal ability to perform an activity at a required level (“instrumentality”) and their belief about the probabilistic association between performance and outcome (“expectancy”). Perceived personal control arguably acts on both of these beliefs. Given that base reward likelihood per se can also affect the subjective value of actions, could the increased subsequent persistence of participants who underwent a high-density condition during the IoC induction phase have resulted simply out of a stronger task-reward association, rather than enhanced perceived control? Such an alternative explanation seems unsatisfactory in relation to the mediation results of Experiment 1, which confirmed that persistence was determined by evoked perceived control rather than the reward density experience itself. While we did not find a significant mediation effect of control ratings in Experiment 2, it still seems unlikely that increased persistence during the competition resulted simply from a higher task-reward association obtained in the IoC induction phase, because during that phase, lightbulb illuminations were not rewarded and participants were unaware that they would later compete and receive financial rewards. Instead, we argue that a conceivable alternative mechanism for our findings is that induced control enhanced participants’ feeling of competence, which in turn is argued to foster intrinsic motivation (Deci & Ryan, 2000; Ryan & Deci, 2000, 2007). Our observation that participants who experienced the high-density condition during the IoC induction phase subsequently predicted having higher control over the task than their opponents indeed suggests that perceived personal control and competence were closely entwined. A competence-related boost in intrinsic motivation could arguably have carried participants over the depletion of extrinsic rewards, at least for some time. A third theoretical position consistent with our findings states that perception of control is inherently rewarding and thereby motivates behavior after outcome value is diminished (Eitam et al., 2013; Karsh et al., 2016; Nafcha et al., 2016; White, 1959). Such an intrinsic motivational value has also been theorized for the neighboring concept of autonomy, which refers to feeling in control over one’s actions rather than feeling pressured (Ryan & Deci, 2000, 2007). Previous studies could demonstrate that autonomy-enhancing interventions − which for instance provided (incidental) choices over the task feedback, order of exercises, or the name of an avatar − enhanced questionnaire measures of intrinsic motivation, willingness to engage in a task (again) and time spent on task (for reviews, see Patall et al., 2008; Wulf & Lewthwaite, 2016). Future research might test the effect of perceived personal control on isolated measures of intrinsic motivation.

Independent of the precise underlying mechanism, our experiments provide strong empirical support for our proposition that augmentation of perceived control will enhance persistence and motivation in clinical and non-clinical training settings. One highly suitable application field would be neurorehabilitation, where patients with acquired brain injury have to relearn hitherto mastered motor and cognitive skills through high-intensive training (Knecht et al., 2016), yet very often cut their training sessions short and spend a lot of time being inactive (Tyson et al., 2015; West & Bernhardt, 2012). Furthermore, despite having some objective control over their training outcome and rehabilitation success, these patients typically perceived their control as being very low (Jones, Mandy, & Partridge, 2009; Lindmark, Wahlberg, & Fugl-Meyer, 2003). This lack of feeling in control is thought to impede their long-term recovery and physical functioning (Bonetti & Johnston, 2008; Johnston, Pollard, Morrison, & MacWalter, 2004; Schröder et al., 2007). Questionnaire-based studies could further link physical activity levels and exercise adherence in both the healthy and the patient populations to control-related beliefs, including expectancy of personal success, perceived competence, and self-efficacy (Petter, Blanchard, Kemp, Mazoff, & Ferrier, 2009; Prince et al., 2016; Sniehotta, Scholz, & Schwarzer, 2005). Our results suggest that adding IoC-inducing features (e.g., high frequency of positive feedback, framing failures as near-miss-like “almost successes”) to neurorehabilitative training sessions would entice patients to exercise for longer and thereby increase training outcomes. It is even conceivable that evoked illusory control would accelerate training-induced learning. Previous research has shown that, in healthy volunteers, motor skill learning can be enhanced by increasing the expectancy of success through selective positive performance (Chiviacowsky & Wulf, 2007; Widmer, Ziegler, Held, Luft, & Lutz, 2016), setting relatively easy criteria for good performance (Chiviacowsky, Wulf & Lewthwaite, 2012; Palmer, Chiviacowsky & Wulf, 2016), and through fabricated positive social comparison (Gonçalves, Cardozo, Valentini, & Chiviacowsky, 2018; Lewthwaite & Wulf, 2010; Wulf, Chiviacowsky, & Cardozo, 2014; Wulf, Chiviacowsky, & Lewthwaite, 2012). This superior learning has been theorized to arise due to a resulting higher success expectancy strengthening goal-action coupling and promoting more effective neural connections, potentially through dopaminergic signaling (Wulf & Lewthwaite, 2016). Arguably, an enhancement of success expectancy due to induced illusory control could tap into the same mechanisms. At the same time, we want to emphasize that IoC applications, particularly in clinical contexts, need to be done with care, as evoking unrealistic expectations about patients’ abilities could be counteractive and lead to decreased rather than increased motivation in the long term. Thus, we suggest that potential future applications of IoC effects in such settings and patients should be restricted to restoring patients’ perceived control to objective levels, rather than aiming to inflate control above reality.

In contrast to persistence, bets placed in the competition of Experiment 2 showed no systematic effect of induced illusory control or overconfidence. Similarly, another recent experiment found that betting on a laboratory gambling task increased in response to streaks of losses but was not related to confidence ratings (Studer, Limbrick-Oldfield, & Clark, 2015). Together these results suggest that betting is primarily governed by preceding outcomes rather than perceived control.

In conclusion, the current experimental research confirms that perceived personal control during an activity can be enhanced through external measures and reveals that such inflation of perceived control prolongs persistence under diminishing returns and in a rigged social competition, that is to say when rewards fade and things get difficult. These findings empirically confirm the long-held theory that our feeling of control determines motivation, and provide a strong argument for employing IoC-inducing features to overcome quitting and motivation failures in rehabilitation, physical activity interventions, and other real-life training settings. Future research might test whether, in addition to boosting immediate persistence as demonstrated here, induced illusory control can also support long-term maintenance of exercise behavior and even improve motor learning.

## Electronic supplementary material

ESM 1 (DOCX 77 kb)

## Footnotes

1 Using a choice between two active responses, rather than between action and non-action as prior research (Alloy & Abramson, 1979; Gillan et al., 2014; Tobias-Webb et al., 2017), ensured that our IoC induction would not be confounded by the rate of active responding (Benvenuti, de Toledo, Simões, & Bizarro, 2017; Hannah & Beneteau, 2009; Yarritu, Matute, & Vadillo, 2014).

2 Additional supplementary moderation analyses performed after square transformation for Experiment 1 (to reduce positive skew) and log transformation for Experiment 2 (to reduce moderate negative skew) provided equivalent results to those reported in the following (see Tables S2 and S3 in Supplementary Material for details).
