# Supplementary material for: Inducing illusory control ensures persistence when rewards fade and when others outperform us
Source: Psychon Bull Rev. 2020 May 18;27(4):809–18. doi: 10.3758/s13423-020-01745-4 (PMC7399668; doi:10.3758/s13423-020-01745-4)
Supplement: Supplementary file 1 — (DOCX 77 kb) [file 13423_2020_1745_MOESM1_ESM.docx]

**SUPPLEMENTARY MATERIAL FOR**

**“Inducing illusory control ensures persistence when rewards fade and when others outperform us”**

by

Bettina Studer, Shawn Geniole, Maike L. Becker, Christoph Eisenegger, Stefan Knecht

**SUPPLEMENTARY METHODS**

**Experiment 1: Exploratory analyses of potential effects of biological sex**

Given that only males were used in experiment 2 due to this experiment being run in the context of a larger protocol examining links between male sex hormones, cognition, and behavior, we conducted an exploratory analysis of potential effects of sex upon IoC induction and persistence in Experiment 1. To do so, we added sex as a covariate to the mixed-repeated measures ANOVA of control ratings during the IOC induction phase and conducted a robust regression model assessing whether sex modulated the relationship between induced illusory control (ratings of perceived control at the end of the IoC induction phase) and persistence (number of trials completed).

**Experiment 2: Exploratory analyses of personality predictors of perceived control**

To explore potential personality factors that could account for variability in (within-condition) ratings of control, we administered the following self-report questionnaires: the shortened version of the Grit Scale (Duckworth, Peterson, Matthews, & Kelly, 2007) to quantify trait perseverance and consistency of interest, the General Self-Efficacy Scale (Schwarzer & Jerusalem, 2010) to measure trait self-efficacy, the Life Orientation Test-Revised (Scheier, Carver, & Bridges, 1994) to assess trait optimism and pessimism, and the Prestige-Dominance Scale (Cheng, Tracy, & Henrich, 2010) to determine trait prestige and dominance. For each of these trait measures, a robust regression model with the outcome variable average rating of control during the *IoC induction phase* and the predictors trait measure score, reward density condition (high or low, experimentally assigned) and the interaction between trait and density condition was calculated with the R robustbase package (Maechler et al., 2018).

**SUPPLEMENTARY RESULTS**

**Development of perceived control over the IOC induction phase (Experiments 1 and 2)**

As reported in the main text, the ANOVAs of ratings of control during the IoC induction phases in Experiments 1 and 2 found significant reward density group x block interactions, Exp 1: *F*(4, 134) = 3.427, *p* = .011, *η^2^* = .093, Exp 2: *F*(5,485) = 3.21, *p* = .007, ηp² = .03. In Experiment 1, control ratings of the *medium density* and *low density* groups declined over the three trial blocks, *F*(2,46) = 4.573, *p* = .016, *η^2^* = .172 and *F*(2,46) = 2.955, *p* = .062, *η^2^* = .114, respectively, whereas the ratings of the *high density* group remained stable, *F*(2, 46) = 1.00, *p* = .376, *η^2^* = .044, such that the difference between perceived control of the *high density* versus the *medium* and *low density* groups increased across the IoC induction phase, see Figure S1A. In contrast, in Experiment 2, the difference between the control ratings of the *high* versus *low density* groups decreased slightly across the six blocks of the *IoC induction phase* (see Figure S1B for ratings in 1^st^ and last blocks).



**Figure S1. Ratings of control in different blocks of the IoC induction phase.**

Average control ratings of the *low* (black lines), *medium* (green lines) and *high* (blue lines) *density condition groups* after each of the three blocks of the IoC induction phase in Experiment 1 (A), and after the first and last block in Experiment 2 (B) are displayed. Error bars represent SEM.

**Experiment 1: Influences of sex upon perceived control and persistence**

No systematic effect of sex was found upon perceived control during the IoC induction phase (main effect of sex: *F* (2, 64) = 0.560, *p* = .456, *η_p_^2^* = .009, sex x reward density group: *F* (2, 64) = 0.276, *p* = .760, *η_p_^2^* = .009, block x sex: *F* (2, 128) = 0.171, *p* = .843, *η_p_^2^* = .003, block x reward density group x sex: *F* (4, 128) = 1.365, *p* = .250, *η_p_^2^* = .041) and all other effects remained qualitatively unchanged from those reported in the manuscript when sex was added as a covariate to the mixed-repeated measures ANOVA of control ratings. Sex also did not significantly modulate the relationship between evoked illusory control and persistence (B = 0.22 [-0.33, 1.38], t(66) = 0.378, p = 0.71) or have an independent effect upon persistence (B = -6.55 [-34.27, 21.25], t(66) = -0.463, p = 0.65).

**Experiment 2: Personality influences upon perceived control**

The exploratory regression analyses testing whether within-condition variability in perceived control during the *IoC induction phase* can be related to individual differences in personality traits found a significant effect for trait self-efficacy, *t*(93) = 2.283, *p* = .025, trait perseverance, *t*(95) = 2.557, p = .012, and trait consistency of interest, *t*(93) = 2.655, *p* = .009, with higher trait scores predicting higher ratings of control. No significant influence was found for the other explored traits (all *p* > .13, see Table S4 for full results).

**SUPPLEMENTARY TABLES**

**Table S1**. Exp. 1: Comparison of demographic and questionnaire data across the three experimental groups.

| Group | *low density* | | *medium density* | | *high density* | | *Group Effect* | |
| --- | --- | --- | --- | --- | --- | --- | --- | --- |
|  | *M /*  *n ♂* | *SD /*  *n ♀* | *M /*  *n ♂* | *SD /*  *n ♀* | *M /*  *n ♂* | *SD /*  *n ♀* |  |  |
| *Age* | 49.83 | 16.19 | 51.13 | 15.03 | 51.70 | 15.13 | *F*(2,69) = .09 | *p* = .914 |
| *Education* | 15.75 | 4.16 | 14.70 | 4.97 | 15.50 | 4.35 | *F*(2,69) = .15 | *p* = .707 |
| *Gender* | 13 | 11 | 7 | 16 | 9 | 14 | *Χ^2^*(2) = 2.80 | *p* = .247 |
| *AES Score* | 42.83 | 7.17 | 42.61 | 10.64 | 42.30 | 7.63 | *F*(2,69) = .02 | *p* = .978 |
| *BDI Score* | 8.92 | 9.93 | 10.35 | 11.780 | 7.35 | 5.85 | *F*(2,69) = .57 | *p* = .567 |

Note: Education stands for years of education, AES Score indicates total score on the German translation of the Apathy Evaluation Scale (Lueken et al., 2006), BDI Scores indicates total scores on the Beck’s Depression Inventory-II (Beck, Steer, & Brown, 1996).

**Table S2**. Exp. 1: Results of mediation analysis with square-transformed persistence measure

|  | *Β* | *SE* | *95% CI* | *z* | *p* |  |
| --- | --- | --- | --- | --- | --- | --- |
| indirect effect  (condition -> control -> persistence) | 3715.81 | 1415.99 | [940.53, 8537.55] | 2.63 | .009 |  |
| condition -> perceived control | 11.55 | 3.22 | [5.233, 17.86] | 4.13 | <.0001 |  |
| perceived control -> persistence | 321.85 | 76.86 | [171.21, 472.49] | 4.19 | <.0001 |  |
| direct effect  (condition -> persistence) | 601.92 | 2356.38 | [-4016.52, 5220.35] | 0.26 | .798 |  |
| R^2^ = .241 | |  |  |  |  |  |

Note: Prior to this analysis, the persistence measures ‘number of completed trials’ was square-transformed to reduce positive skew (skewness untransformed = -1.16, transformed = -0.93).

**Table S3**. Exp. 2: Results of mediation analysis with log-transformed persistence measure

|  | *Β* | *SE* | *95% CI* | *z* | *p* |  |
| --- | --- | --- | --- | --- | --- | --- |
| indirect effect  (condition -> control -> persistence) | 0.056 | 0.09 | [-0.07, 0.13] | 0.63 | .53 |  |
| condition -> perceived control | 29.78 | 0.09 | [0.30, 0.65] | 5.37 | <.0001 |  |
| perceived control -> persistence | 0.002 | 0.003 | [-0.004, 0.01] | 0.65 | .52 |  |
| direct effect  (condition -> persistence) | 0.42 | 0.20 | [0.03, 0.82] | 2.10 | .04 |  |
| R^2^ = .241 | |  |  |  |  |  |

Note: Prior to this analysis, the persistence measures ‘number of completed trials’ was square-transformed to reduce positive skew (skewness untransformed = 1.15, transformed = -0.77).

**Table S4.** Exp. 2: Results from exploratory robust regression analyses examining the influence of candidate personality traits upon perceived control during the *IoC induction phase.*

| Dominance Model | *B* | *SEM* | *t*(95) | *p* |
| --- | --- | --- | --- | --- |
| ***high density condition*** | ***40.278*** | ***4.119*** | ***9.778*** | ***<.001*** |
| *Dominance* | *2.809* | *1.857* | *1.513* | *.134* |
| *High density condition x Dominance* | *-0.772* | *3.690* | *-0.209* | *.835* |
| Prestige Model | *B* | *SEM* | *t*(95) | *p* |
| ***High density condition*** | ***40.277*** | ***4.570*** | ***8.813*** | ***<.001*** |
| *Prestige* | *0.513* | *2.305* | *0.223* | *.824* |
| *High density condition x Prestige* | *5.079* | *4.774* | *1.064* | *.290* |
| Grit-Interest Model | *B* | *SEM* | *t*(93) | *p* |
| ***High density condition*** | ***41.169*** | ***3.789*** | ***10.865*** | ***<.001*** |
| ***Grit-Consistency of Interest*** | ***5.410*** | ***2.038*** | ***2.655*** | ***.009*** |
| *High density condition x Grit-Interest* | *5.103* | *4.106* | *1.243* | *.217* |
| Grit-Persistence Model | *B* | *SEM* | *t*(95) | *p* |
| ***High density condition*** | ***39.304*** | ***4.096*** | ***9.596*** | ***<.001*** |
| ***Grit-Persistence*** | ***4.930*** | ***1.928*** | ***2.557*** | ***.012*** |
| *High density condition x Grit-Persistence* | *4.148* | *3.841* | *1.080* | *.283* |
| LOT-R Optimism Model | *B* | *SEM* | *t*(93) | *p* |
| ***High density condition*** | ***40.574*** | ***4.462*** | ***9.092*** | ***<.001*** |
| *LOT-R Optimism* | *1.135* | *2.058* | *0.551* | *.583* |
| *High density condition x LOT-R Optimism* | *-4.182* | *4.103* | *-1.019* | *.311* |
| LOT-R Pessimism Model | *B* | *SEM* | *t*(96) | *p* |
| ***High density condition*** | ***39.252*** | ***4.357*** | ***9.009*** | ***<.001*** |
| *LOT-R Pessimism* | *-2.556* | *2.129* | *-1.201* | *.233* |
| *High density condition x LOT-R Pessimism* | *4.912* | *4.218* | *1.165* | *.247* |
| LOT-R Total Model | *B* | *SEM* | *t*(91) | *p* |
| ***High density condition*** | ***40.053*** | ***4.397*** | ***9.109*** | ***<.001*** |
| *LOT-R Total* | *1.839* | *2.081* | *0.883* | *.379* |
| *High density condition x LOT-R Total* | *-4.407* | *4.113* | *-1.071* | *.287* |
| Self-Efficacy Model | *B* | *SEM* | *t*(93) | *p* |
| ***High density condition*** | **38.624** | **4.067** | **9.496** | **<.001** |
| ***Self-Efficacy*** | **3.712** | **1.626** | **2.283** | **.025** |
| ***High density condition x Self-Efficacy*** | **7.447** | **3.291** | **2.263** | **.026** |

Note: The averaged rating of control during the *IoC induction* *phase* served as the outcome measure. *n* and *df* varied across models because of variable missing data in the questionnaire measures. The *low density* condition served as a reference. Predictors that reached statistical significance are printed in bold. Calculated with the robustbase R package.

**SUPPLEMENTARY REFERENCES**

Beck, A. T., Steer, R. A., & Brown, G. K. (1996). Beck depression inventory-II. *San Antonio, 78*(2), 490-498.

Cheng, J. T., Tracy, J. L., & Henrich, J. (2010). Pride, personality, and the evolutionary foundations of human social status. *Evolution and Human Behavior, 31*(5), 334-347.

Duckworth, A. L., Peterson, C., Matthews, M. D., & Kelly, D. R. (2007). Grit: perseverance and passion for long-term goals. *Journal of Personality and Social Psychology, 92*(6), 1087-1101. doi:10.1037/0022-3514.92.6.1087

Lueken, U., Seidl, U., Schwarz, M., Völker, L., Naumann, D., Mattes, K., . . . Schweiger, E. (2006). Die Apathy Evaluation Scale: Erste Ergebnisse zu den psychometrischen Eigenschaften einer deutschsprachigen Übersetzung der Skala. [Psychometric Properties of a German Version of the Apathy Evaluation Scale]. *Fortschr Neurol Psychiatr, 74*(12), 714-722. doi:10.1055/s-2006-932164

Maechler, M., Rousseeuw, P., Croux, C., Todorov, V., Ruckstuhl, A., Salibian-Barrera, M., . . . di Palma, M. A. (2018). Robustbase: Basic Robust Statistics R Package Version 0.93-0. Retrieved from <http://CRAN.R-project.org/package=robustbase>

Scheier, M. F., Carver, C. S., & Bridges, M. W. (1994). Distinguishing optimism from neuroticism (and trait anxiety, self-mastery, and self-esteem): a reevaluation of the Life Orientation Test. *Journal of Personality and Social Psychology, 67*(6), 1063.

Schwarzer, R., & Jerusalem, M. (2010). The general self-efficacy scale (GSE). *Anxiety, Stress, and Coping, 12*, 329-345.
